# Research on the influencing factors of using health science popularization short videos on the self-management behavior of older adult patients with chronic diseases

**DOI:** 10.3389/fpubh.2026.1720548

**Published:** 2026-01-21

**Authors:** Yilin Zhang, Miao Yu, Zhenghan Gao

**Affiliations:** School of Humanities and Foreign Languages, Qingdao University of Technology, Qingdao, China

**Keywords:** healthscience popularization short videos, influencing factors, older adult patients with chronic diseases, self-management behavior, structural equation modeling

## Abstract

**Background:**

The global prevalence of chronic diseases among the older adults population continues to rise, emerging as a core issue affecting the health and quality of life of older adults. This trend is particularly pronounced in China, which has entered a phase of deep aging society. With the rapid development of the internet, digital media technologies—represented by health science popularization short videos, offer new possibilities for self-health management in older adults.

**Methods:**

Utilizing data collected via face-to-face electronic questionnaires from 2024 to 2025 (*N* = 833), this study employed multivariable logistic regression model to analyze the role of various sociodemographic factors influencing self-management behavior following the use of health science popularization short videos among older adult patients with chronic diseases. Structural equation model (SEM) was used to analyze the mechanisms underlying these self-management behavior, incorporating descriptive statistics, regression analysis, and mediation testing.

**Results:**

The results indicate that watching health science popularization short videos had a significant positive effect on the self-management behavior of older adults patients with chronic conditions. Self-perceived level, medical compliance, and family caregiving level were identified as significant factors influencing both trust in the video content and self-management behavior. Content trustworthiness mediated this relationship and also directly influenced self-management behavior. Significant differences were observed based on “urban–rural location” and “extent of media exposure”; however, no significant behavioral differences were found by “gender,” and “education level” and “income” did not significantly affect content trustworthiness.

**Conclusion:**

This study treats digital media use as a key research variable. The findings suggest that in promoting the adaptation of digital health services for older adults, it is essential to strengthen the credibility of health science content and address the diverse needs of older adult populations across different regions and with varying media usage habits. Furthermore, by enhancing self-efficacy and strengthening family support, among other dimensions, more targeted health behavior intervention strategies should be developed. This approach will help to fully leverage the positive role of digital media in improving health management among older adults.

## 1 Introduction

Against the backdrop of a rapidly aging global population, health issues among older adults have become a critical focus in public health. The global population aged 60 and above continues to grow, a trend paralleled by a sharp rise in the prevalence of chronic non-communicable diseases (hereafter chronic diseases). The World Health Organization estimates over 2 billion people globally live with chronic diseases; approximately 60% of individuals aged 60 and above have at least one chronic condition, making these diseases a leading cause of mortality worldwide (1). According to the concurrent data from the National Health Commission of China, the prevalence rate of chronic diseases in China has reached 35–45%, and the number of chronic disease patients with self-management behaviors is about 500 million to 600 million. Among them, there are over 180 million older adult patients with chronic diseases aged 60 and above; 75.8% of older adults have at least one chronic disease, and 43% experience multimorbidity (the co-occurrence of two or more chronic conditions) (2). Chronic diseases account for over 80% of all deaths and contribute to 70% of the total disease burden in China (3–5). However, control rates for chronic diseases among older adult patients in China are markedly low. Only 14.6% of hypertensive patients achieve controlled blood pressure, with this figure falling below 10% for those aged 65–75. The control rate for diabetes is approximately 37.3%. Management of chronic obstructive pulmonary disease is challenged by low disease awareness and underutilization of pulmonary function testing (6–8). This situation exacerbates health risks for older adults within an aging society, places severe strain on family caregiving capacities and the allocation of medical resources, and reveals significant limitations of traditional management models. These challenges underscore the urgent need to transition the management of chronic diseases in older adults from a paradigm centered on “medical intervention” to one that incorporates “digital participation.”

Scientific research indicates that approximately 80% of chronic diseases are preventable through lifestyle modifications, including balanced diets, regular physical activity, smoking cessation, alcohol moderation, and maintaining a healthy body weight (9). Technology empowerment in the digital era presents new opportunities for addressing this public health challenge. According to the 56th Statistical Report on China’s Internet Development, the population of internet users aged 60 and above reached 161 million in 2025. A substantial proportion of these older adults are active on popularization short video platforms for entertainment, social connection, and information acquisition, establishing these platforms as a core component of their digital engagement (10). In recent years, health science popularization short videos on social media platforms (e.g., TikTok, Instagram, WeChat) have emerged as a significant channel for health information. Leveraging advantages such as fragmented accessibility, visual presentation, and low technical barriers, these videos align with the cognitive preferences of older adults. They facilitate access to practical knowledge on disease prevention, daily self-care, and medication adherence, thereby supporting improvements in health lifestyles among older adult patients with chronic diseases (11). It can be argued that health science popularization short videos have reshaped the health information environment for older adults, simultaneously fostering a theoretical research framework examining the pathway from “media usage” to “health cognition” and ultimately to “behavioral change” in self-management behavior.

Existing research on chronic diseases in older adults has predominantly resided within public health and clinical medicine. While this has established mature clinical pathways for chronic disease management, it has largely overlooked the influence of digital tool utilization in new media environments on the self-management behavior of older adult patients with chronic diseases. Furthermore, it has seldom integrated health communication into a comprehensive chronic disease management research framework. Concurrently, research in journalism and communication studies has increasingly focused on novel media like popularization short videos. However, it often concentrates on the dissemination status, content features of health-related videos, or the usage behaviors of generalized audiences. This body of work frequently neglects to make “older adult patients with chronic diseases”—a group characterized by high needs and high vulnerability—its central focus, resulting in a lack of mechanistic investigation into how popularization short videos impact this demographic’s self-management behavior. This study aims to bridge these disciplinary gaps by integrating perspectives from public health, clinical medicine, and health communication. It seeks to examine the health behaviors and digital practices of older adult patients with chronic diseases within social media environments, thereby addressing the practical needs for digital services in an aging society under the strategic context of “Healthy China Action (2019–2030).”

Given the aforementioned research background and theoretical gaps, fostering effective self-management behavior is considered particularly crucial for disease control among older adult patients with chronic diseases. Chronic diseases are characterized by their prolonged duration, tendency for recurrence, and multi-system involvement. Consequently, consistent and standardized self-management is fundamental for older patients to maintain stable health conditions, reduce complication risks, and enhance their quality of life. Self-management behavior (hereafter SMB) refers to a comprehensive set of actions wherein individuals, guided by healthcare professionals, proactively monitor, intervene, and maintain their own health—particularly in managing chronic conditions—through cognitive, behavioral, and emotional regulation. This aims to prevent disease progression, improve quality of life, and promote positive health outcomes (12). In medical research, SMB is often categorized into three dimensions: disease-specific management, lifestyle management, and emotional/cognitive management. This study integrates the media usage patterns of older adult patients with chronic diseases with the communication features of popularization short video platforms—such as active information seeking, social interaction, emotional dependence, media literacy, usage intention, and effect evaluation. Using a quantitative research approach, it investigates the extent and influencing factors of self-management behavior after older chronic disease patients use health education popularization short videos. Specifically, the research will focus on: the ability to discern information during active seeking; the effect of family support in social interactions; the role of emotional dependency in promoting behavioral persistence; and the moderating effect of media literacy on the transformation of health cognitions. This research holds significant value for promoting more optimal health outcomes among older chronic disease patients in China.

## 2 Theoretical and hypothesis support

### 2.1 Research on the health belief model, behavior change theories, and core individual influencing factors

The Health Belief Model (HBM), proposed by Hochbaum and Rosenstock in their 1952 book Health Belief Model to explain the decision-making process of individuals’ health behaviors. It posits that health behaviors are influenced by the combined effect of perceived susceptibility, perceived severity, perceived benefits, and perceived barriers (13). Since its introduction into chronic disease self-management research in the 1990s, substantial evidence has demonstrated the HBM’s particular applicability among older adult populations, effectively explaining variations in their Self-management behavior. Ibrahim et al. found that “self-perceived” factors such as “perceived severity” and “perceived benefits” were significant predictors of vaccination behavior in older adults. Sharifirad et al., through comparative age studies, found that the HBM holds stronger predictive power for the health behaviors of older adult patients with diabetes. Xie et al. demonstrated that health education via short videos could significantly improve blood glucose levels, adherence to glucose monitoring, and self-efficacy among older adult patients with diabetes. Based on this, the present study consolidates the four dimensions of the HBM into a measurable construct termed the “Self-Perception Level” (SPL), positing that enhancing the SPL of Older adult patients with chronic diseases is a key mechanism for improving their Self-management behavior. However, the traditional HBM focuses on individual perceptions and beliefs, with insufficient consideration for the external conditions and comprehensive capabilities required for behavior to occur. To address this limitation, this study adopts the COM-B (Capability, Opportunity, Motivation-Behavior) system model of behavior change, proposed by Professor Susan Michie and colleagues, as its macro framework. This model posits that any behavior results from the interaction of three components: Capability, Opportunity, and Motivation (14). Within this framework, the “Self-Perception Level” emphasized by the HBM can be precisely situated as a core component of “Motivation.” Simultaneously, the act of viewing short videos itself may influence the psychological and physical capability of older adult patients. Furthermore, support and care from family provide crucial social and physical opportunity. Thus, the HBM receives a more systematic interpretation within the COM-B framework.

An individual’s self-perception level serves as a significant antecedent variable in their assessment of health information credibility. Older adult patients with chronic diseases who have a higher SPL more actively filter information and demonstrate a greater tendency to trust scientific content that aligns with their specific health needs. Research on patients with diabetes indicates that the influence of self-perception within the health belief framework on self-management behavior implies a mediating role of trust in educational information. During the COVID-19 pandemic, self-perception indirectly strengthened vaccination intention among adults by enhancing trust in professional sources. This evidence corroborates the efficacy of “trust” as a form of “psychological motivation” in generating and altering individual behavior. Based on the above analysis, the following hypotheses are proposed:

*H1*: The self-perception level of older adult patients with chronic diseases has a positive effect on the perceived content trustworthiness of health science popularization short videos.

*H2*: There is a positive correlation between the self-perception level of older adult patients with chronic diseases after using health science popularization short videos and their self-management behavior.

In chronic disease management practice, medical compliance (hereafter MC, encompassing medication adherence, follow-up visits, and treatment plan execution) is widely regarded as the most core and observable dimension of self-management behavior, directly influencing disease control outcomes and long-term patient prognosis. A bidirectional reinforcement mechanism likely exists between medical compliance and information trustworthiness. On one hand, trust is a significant antecedent of medical compliance. On the other hand, when patients act upon trusted health information and receive positive feedback, this successful experience reciprocally reinforces their trust in the original information source, creating a virtuous cycle. Compared to conventional care, mobile applications serve as effective interventions for improving medication adherence among adult patients with chronic diseases. Education based on the Health Belief Model (HBM) has been shown to improve medication and check-up adherence among patients with hypertension and diabetes by enhancing trust. Within the context of health science popularization short videos, an improvement in medical compliance likely indicates comprehensive enhancement of self-management behavior across multiple aspects, such as diet and exercise. Therefore, this study proposes the following hypotheses:

*H3*: The medical compliance of older adult patients with chronic diseases has a positive effect on the content trustworthiness of health science popularization short videos.

*H4*: There is a positive correlation between the medical compliance of older adult patients with chronic diseases after using health science popularization short videos and their self-management behavior.

### 2.2 Research on users’ self-management behavior in family care and media usage

Beyond individual factors, family caregiving level (FCL), as a core component of the social support system, plays a significant role in shaping the health outcomes of older adult patients with chronic diseases. Social Support Theory posits that the material, emotional, and informational support individuals receive through their social networks is crucial for their physical and mental wellbeing (15). In this study, the instructive behavior whereby younger family members assist older generations with digital access and usage is termed “digital feedback,” with the family serving as its primary setting. The digital skill support (e.g., guiding elders to use smartphones for health information searches and assessing video content quality) and emotional support (e.g., encouraging elders to try online consultations) provided by family members can influence the trust judgments of older adult patients with chronic diseases regarding health information. This support may even directly affect their digital health literacy—the ability to use information technology to improve health outcomes—which is a key antecedent influencing their engagement in digital health behaviors (16). Such intergenerational digital feedback can effectively bridge the digital divide for the older population, significantly alleviate stress and technophobia related to their health conditions, and promote the adoption of adaptive health behaviors (17, 18). This also illustrates that family caregiving level, acting as an enabling factor within behavior change theories, can enhance older adults’ psychological readiness and technical capability to utilize digital health tools like short videos through the mechanism of digital feedback.

Research indicates that media intervention with intergenerational support—where younger family members assist older adults in verifying information authenticity and comprehending digital content—can enhance older adults’ technology perception and trust, thereby increasing their willingness to engage in online medical consultations (19, 20). Patients with diabetes who experience a high family caregiving level demonstrate better self-management behavior, including dietary control, physical exercise, and blood glucose monitoring (21). Among patients with coronary heart disease, family caregiving level not only directly and positively predicts self-management capacity but also indirectly promotes self-management by enhancing patients’ self-efficacy (22). Through ongoing encouragement and collaborative practice from children or grandchildren, family caregiving level serves as a significant external factor in establishing older adults’ trust in digital health information. Thus, the following hypotheses are proposed:

*H5*: The family caregiving level of older adult patients with chronic diseases has a positive effect on the content trustworthiness of health science popularization short videos.

*H6*: There is a positive correlation between the family caregiving level of older adult patients with chronic diseases after using health science popularization short videos and their self-management behavior.

### 2.3 Research on content trust mechanisms in health communication

Health communication research increasingly focuses on variables influencing audience psychology, with trust recognized as a critical bridge between information delivery and behavior change (23). Within digital health interventions, the concept of “engagement” is pivotal. Trust does not inevitably lead to behavior change; rather, change predominantly stems from deep engagement across behavioral, cognitive, and emotional dimensions between the user and the content. Trust in the content is a prerequisite for such deep engagement. When patients trust a video, they are more likely to repeatedly watch, save, or share it (behavioral); to thoughtfully process and remember its key knowledge points (cognitive); and to identify with and resonate emotionally with the health concepts it communicates (emotional). This multi-dimensional engagement ultimately catalyzes the translation of video knowledge into practical self-management behavior. Furthermore, the positive feedback from successful self-management behavior can reinforce the patient’s trust in the original information source, creating a virtuous, reinforcing cycle. Chang et al. noted that content-related indicators and criteria are most frequently used when evaluating health information sources and quality (24). Soroya et al. revealed that perceived credibility mediates the relationship between health-related internet use and disease management behaviors (25). Content trustworthiness (CT) serves as a core mediator, effectively linking an individual’s exposure to media information with subsequent behavioral change. However, other research indicates that older adult patients with chronic diseases, due to cognitive constraints, diminished attention, and information overload, often struggle to verify information accuracy effectively. This can lead to skepticism about the authenticity of online health content and even health information avoidance (26, 27).

On one hand, internal factors such as video production quality, presentation format, the institutional authority of physicians, and informational accuracy directly shape patients’ initial impressions and form the foundation of trust, particularly in chronic disease management. Videos featuring medical experts and clear scientific evidence are perceived as more reliable by patients (28, 29). On the other hand, external factors including patients’ prior knowledge, health literacy, past experiences, and social support also influence their trust judgments. Older patients with higher health literacy are better equipped to discern information quality, while recommendations or endorsements from family and friends can enhance their trust in popularization short video content (30), and vice versa. Based on the above analysis, the following hypotheses are proposed:

*H7*: The content trustworthiness of health science popularization short videos among older adult patients with chronic diseases positively influences their self-management behavior.

*H8*: The content trustworthiness of health science popularization short videos among older adult patients with chronic diseases mediates the relationship between "health science popularization short video usage" and "self-management behavior."

**Figure 1:**
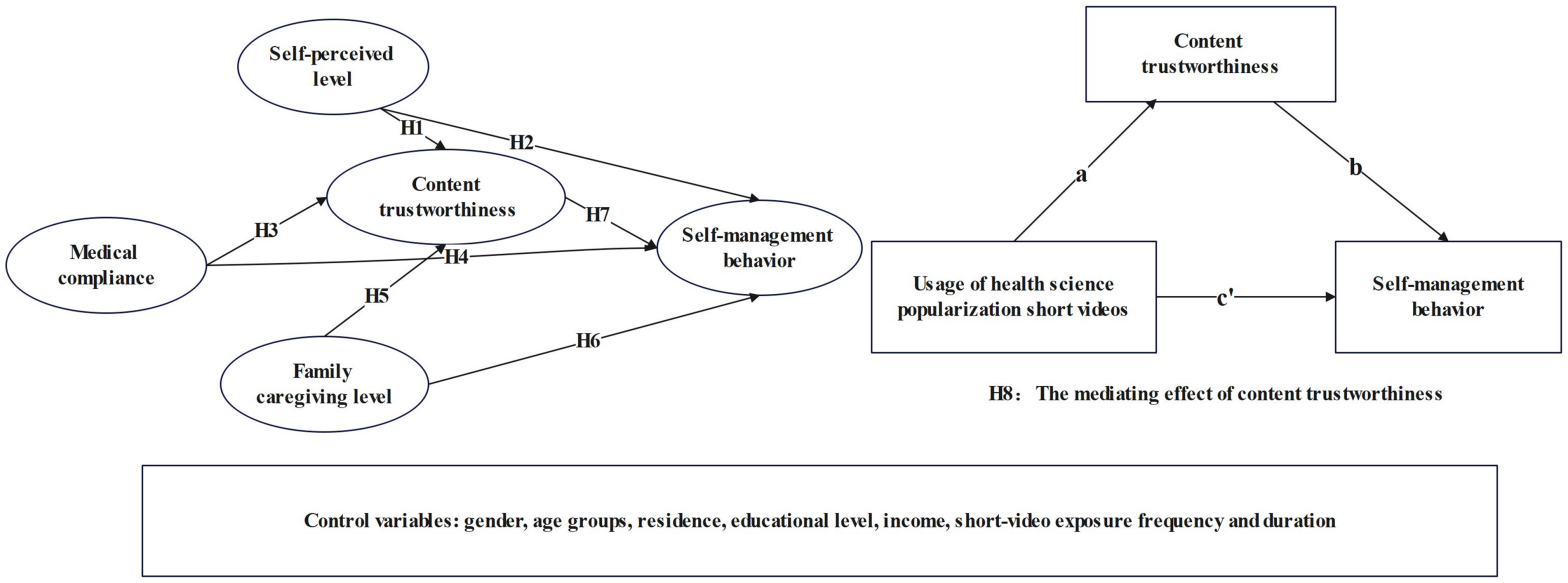
Analytical model of health science popularization short video usage impact on self-management behavior in older adult patients with chronic diseases.

In summary, grounded in the Health Belief Model, Social Support Theory, and health communication effect analysis, this study has formulated eight research hypotheses. An analytical model was ultimately constructed to examine the impact of health science popularization short video usage on the self-management behavior of older adult patients with chronic diseases (see Figure 1). This model specifies self-perception level, medical compliance, and family caregiving level as independent variables; content trustworthiness as the mediating variable; self-management behavior as the dependent variable; and includes eight control variables.

## 3 Methods

### 3.1 Sampling plan and survey instruments

This study employed a questionnaire survey method administered through the professional survey platform “Credamo”^1^. A multi-stage Probability Proportional to Size (PPS) sampling design was implemented to enhance the national representativeness of the sample among older adult patients with chronic diseases. Stage 1: All prefecture-level administrative divisions in China (including prefecture-level cities, municipal districts of direct-controlled municipalities, sub-provincial cities, and autonomous prefectures) served as the primary sampling units. Using the “population aged 60 and above” from the Seventh National Population Census as the measure of size, 25 cities were selected via PPS sampling. These cities demonstrate substantial heterogeneity and national representativeness in terms of aging level, economic development, and medical resource distribution. Stage 2: Within each selected city, stratified sampling was conducted. All medical institutions were first categorized into three strata: community health service centers, Class II Grade A hospitals, and Class III Grade A hospitals. Within each stratum, PPS sampling was then performed using the “annual number of visits by older adult patients with chronic diseases” (data sourced from local health statistical yearbooks or medical insurance billing systems) as the Measure of Size, selecting 3–5 institutions from each stratum. Stage 3: Within each selected institution, the hospital information system (HIS) was used to generate a list of all eligible patients, forming the final sampling frame. To mitigate potential biases associated with simple random sampling and enhance operational feasibility, systematic sampling was employed: a sampling interval k was calculated (k = total patients / required sample size), and patients were selected at this fixed interval from a random starting point until the sample size requirement was met. Inclusion Criteria: (1) Age≥60 years (referencing the *China Aging Development Report*); (2) Diagnosed with a chronic disease according to ICD-10 criteria, with a disease duration≥3 months; (3) Capable of independently using the internet or smart devices; (4) Informed and provided consent to participate. Exclusion Criteria: (1) Patients with cognitive impairment or mental disorders; (2) Lacking basic literacy skills; (3) Those who withdrew from the survey prematurely.

To ensure scientific validity and questionnaire reliability, all measurement items in this study were adapted from established scales and contextually modified to align with the specific research objectives. The survey encompassed five dimensions: self-perception level, medical compliance, family caregiving level, content trustworthiness, and self-management behavior. All items were measured using a 5-point Likert scale, ranging from 1 (“strongly disagree”) to 5 (“strongly agree”). The complete survey instrument is provided in Supplementary material.

### 3.2 Data collection and statistical methods

Data were collected through online questionnaires administered by trained surveyors using a one-on-one guided completion approach. Prior to data collection, the research team conducted detailed discussions on item wording and technical implementation details to ensure methodological rigor and consistency. Accompanied by clinical staff, surveyors explained the study’s purpose and procedures to potential participants. Written informed consent was obtained before questionnaire administration. Each survey session was limited to 30–40 min to prevent fatigue-related response quality degradation.

The study protocol received ethical approval, and privacy protection agreements were established with participating medical institutions, strictly adhering to voluntary participation principles. Chi-square tests were employed to analyze differences in self-management behavior through health popularization short video usage among older adult patients with chronic diseases across various sociodemographic characteristics. Multivariable logistic regression models were used to examine the influence of sociodemographic factors on self-management behavior following health science popularization short video usage. All continuous variables underwent normality testing using SPSS software. Structural equation model (SEM) was conducted using AMOS software, with mediation effects tested through Bootstrap methods. Data were initially processed using Excel 2019 (Microsoft Inc. Washington, DC, USA), analyzed with IBM SPSS Statistics (Ver. 27, IBM Inc. New York, USA), and modeled with AMOS (Ver. 29).

## 4 Data analysis and results

### 4.1 Current status of health short-video usage for self-management among older adult patients with chronic diseases

A total of 976 questionnaires were collected. After excluding responses with logical contradictions or evident response patterns, 833 valid questionnaires were retained, yielding a valid response rate of 85.3%. The sociodemographic characteristics and online participation patterns of the 833 respondents are presented in Table 1.

**Table 1:** Basic characteristics of respondents.

Control variables		*n* (%)	Control variables		*n* (%)
Gender	Male	378(45.4)	Used platforms	Public social media	588(70.6)
	Female	455(54.6)		Official social media	409(49.1)
Age group	60–64	347(41.7)		Content aggregation platforms	276(33.1)
	65–69	181(21.7)		Medical and health self-media	118(14.2)
	70–74	142(17.0)	Content preferences	Exercise, healthy diet, and lifestyle habits	566(67.9)
	75–79	83(10.0)		Mental health and emotional management	367(44.1)
	≥80	80(9.6)		Disease symptoms, diagnosis, and treatment	401(48.1)
Residence	Rural	308(37.0)		Others’ illness experiences and solutions	266(31.9)
	Urban	525(63.0)		Drug information	251(30.1)
Educational level	Primary school or below	230(27.6)		Medical insurance and health policies	209(25.1)
	Junior high school	213(25.6)		Doctor or healthcare institution information	156(18.7)
	Senior high school	185(22.2)		Others	157(18.8)
	Junior college	87(10.4)	Exposure frequency	<1/month	190(22.8)
	Bachelor’s degree or above	118(14.2)		1–3/month	212(25.5)
Income	No fixed income	217(26.1)		1–2/week	172(20.6)
	<1,000	124(14.9)		3–6/week	161(19.3)
	1,000–3,000	186(22.3)		≥1/day	98(11.8)
	3,001–5,000	151(18.1)		<0.5 h	341(40.9)
	≥5,000	155(18.6)	Exposure duration	0.5-1 h	265(31.8)
				1–2 h	143(17.2)
				≥2 h	84(10.1)

**Figure 2:**
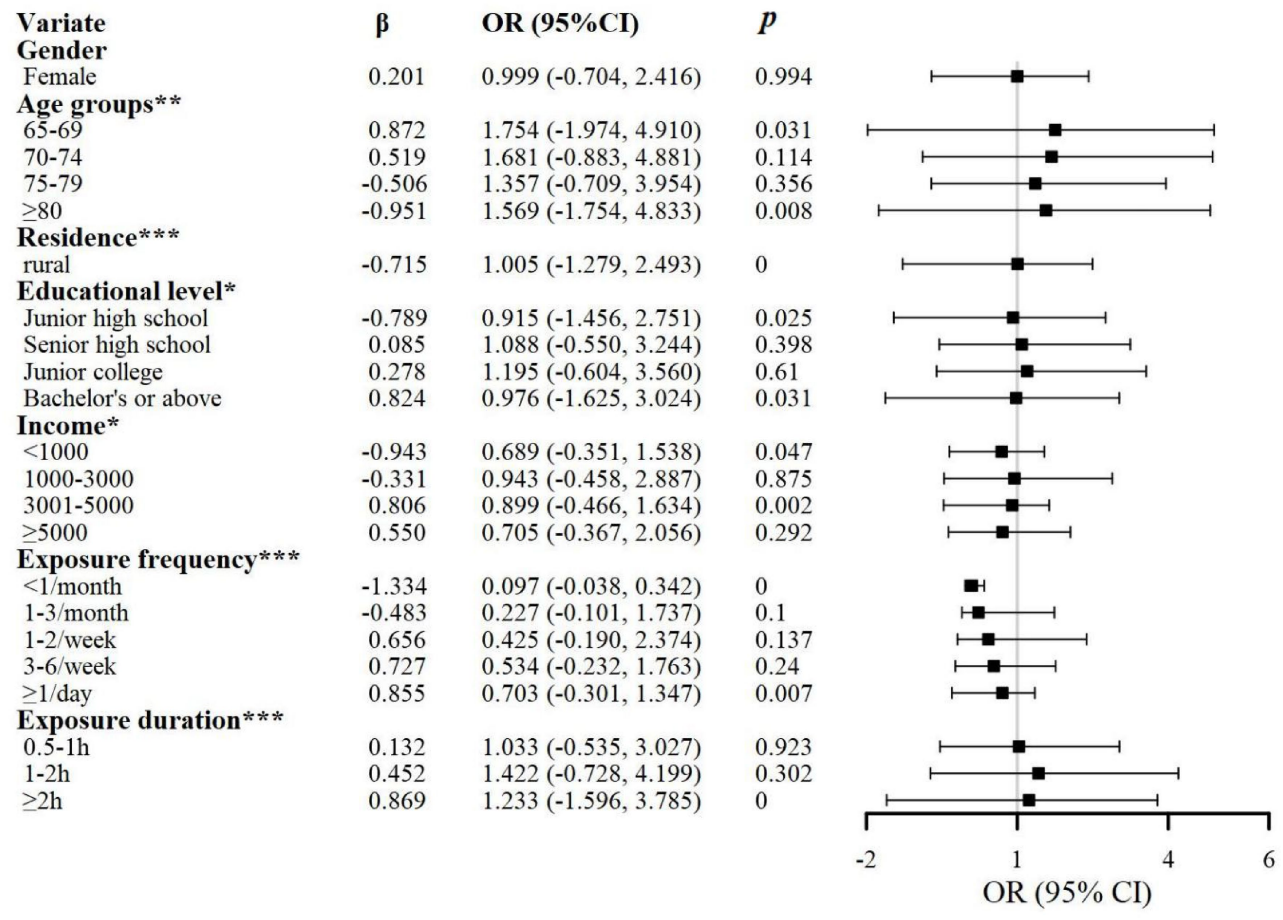
Results of chi-square test and regression analysis of self-management behavior in older adult patients with chronic diseases. *p < 0.05; **p < 0.01; ***p < 0.001.

This study employed a binary dependent variable indicating the presence or absence of self-management behavior (coded: 0 = no, 1 = yes). Based on the Health Belief Model and established scales by Xuan, Lorig, Liu, and Xiao et al. (31–33), we developed a measurement scale for self-management behavior related to health science popularization short video usage among older adult patients with chronic diseases (see Supplementary material). The scale encompasses seven dimensions: physical exercise, cognitive symptom management, physician communication, social support, digital health information acquisition and evaluation skills, digital health information interaction skills, and digital health information application skills, with two additional dimensions—media use intention and communication effect feedback. The scale comprises 21 items with a maximum score of 100. A total score≥60 was defined as indicating “presence of self-management behavior,” while a score of <60 was categorized as “absence of self-management behavior.” A higher score indicates a stronger self-management behavior capability in the patient. Multivariable logistic regression analysis revealed that 435 participants (52.2%) were classified as older adult patients with chronic diseases without self-management behavior, while 398 (47.8%) were classified with self-management behavior. Specific sociodemographic factors associated with reduced self-management behavior included: age≥80 years (*β* = −0.951), rural residence (*β* = −0.715), education level of junior high school or below (*β* = −0.789), monthly household income<1,000 RMB (*β* = −0.943), and watching health science popularization short videos less than once per month (*β* = −1.334). In contrast, factors associated with increased self-management behavior included: age<70 years (*β* = 0.872), bachelor’s degree or higher education (*β* = 0.824), monthly household income of 3,000–5,000 RMB (*β* = 0.806), daily usage of health popularization short videos (*β* = 0.855), and watching duration exceeding 2 h (*β* = 0.869) (see Figure 2). Independent samples *t*-tests and one-way ANOVA results indicated significant differences in self-management behavior among older adult patients with chronic diseases across age, urban–rural residence, media exposure frequency, and exposure duration. However, no significant gender-based differences were observed. Education level and monthly income showed partial differences in self-management behavior but demonstrated no direct effects on content trustworthiness.

### 4.2 Reliability and validity tests

Prior to the formal survey, the questionnaire used in this study strictly followed scale development and validation procedures. First, based on a literature review and theoretical framework, the initial items were drafted. To ensure content validity, five experts in health communication, geriatric nursing, chronic disease management, and psychology were invited to evaluate the relevance, representativeness, and clarity of the questionnaire items in relation to their corresponding theoretical constructs over two rounds. The content validity ratio (CVR) was then calculated, with the content validity index (CVI) for each construct exceeding the acceptable threshold of 0.8. Subsequently, a pilot test was conducted within the target population. Researchers recruited 50 eligible older patients from community health centers for a small-scale pre-survey to preliminarily examine the questionnaire’s reliability and structure. Based on data analysis from the pilot test (Cronbach’s *α* coefficients all above 0.7) and post-interviews, minor revisions were made to the wording and sequencing of the items, resulting in the final version of the questionnaire for large-scale formal investigation.

Using data from the large-scale formal survey, the reliability and validity of the measurement model were rigorously tested. This study employed Cronbach’s *α* coefficient and composite reliability (CR) as reliability indicators. As shown in Table 2, both the Cronbach’s α and CR values for all variables exceeded the acceptable threshold of 0.7, indicating high overall reliability of the scale. For validity assessment, convergent validity was evaluated using factor loadings and average variance extracted (AVE). The AVE values for all variables surpassed the 0.5 benchmark (Table 2), demonstrating satisfactory convergent validity of the measurement items with their respective dimensions. Discriminant validity was assessed by comparing the square roots of the AVE values with the Pearson correlation coefficients between dimensions. As presented in Table 3, the square roots of the AVE for all five dimensions were greater than their correlations with other dimensions, indicating clear discrimination among the five core constructs and demonstrating good discriminant validity in the sample data.

**Table 2:** Reliability and validity analysis.

Variate	Variate(abbreviation)	Cronbach’s Alpha	AVE	CR
Self-perceived level	SPL	0.902	0.592	0.772
Medical compliance	MC	0.740	0.552	0.703
Family caregiving level	FCL	0.733	0.506	0.745
Content trustworthiness	CT	0.862	0.518	0.736
Self-management behavior	SMB	0.796	0.609	0.817

**Table 3:** Ave square root values (Pearson correlation coefficients for each dimension).

Variate	Self-perceived level	Medical compliance	Family caregiving level	Content trustworthiness	Self-management behavior
Self-perceived level	0.769*				
Medical compliance	0.577	0.743*			
Family caregiving level	0.496	0.481	0.711*		
Content trustworthiness	0.350	0.236	0.282	0.720*	
Self-management behavior	0.634	0.642	0.634	0.371	0.780*

*p < 0.05.

## 5 Model construction: factors influencing the self-management behavior of older adult patients with chronic diseases through health science popularization short videos

### 5.1 Selection of structural equation modeling

This study aims to elucidate a complex mechanism involving multiple latent variables and their interactions—specifically, how self-perception, medical compliance, and family caregiving level directly affect patients’ self-management behavior, and how they exert indirect effects through the key psychological variable of content trustworthiness. The core strength of Structural Equation Modeling (SEM) lies in its ability to simultaneously handle measurement and structural models, thereby testing multivariate mechanisms. On one hand, it treats theoretical constructs that cannot be directly observed—such as “self-perception” and “content trust”—as latent variables, estimated through multiple observed indicators, which effectively controls for measurement error. On the other hand, it can test the significance and direction of all hypothesized pathways within an integrated framework and provide various goodness-of-fit indices to evaluate how well the overall theoretical model aligns with the empirical data. This capacity for holistic examination of multi-factor relationships is not offered by traditional regression analysis methods. Compared to SEM, the Structural Causal Model (SCM) typically requires identification strategies such as randomized experiments, natural experiments, or valid instrumental variables. Its core aim is to address counterfactual questions of “what would happen if an intervention were implemented.” However, this study did not introduce external instrumental variables, and it was difficult to identify a completely uncontaminated natural experiment setting or to achieve randomized intervention on the media use behavior of older adult patients with chronic diseases. Therefore, the clear causal identification conditions required by SCM were not met. While Directed Acyclic Graphs (DAG) are useful for clarifying the causal order among variables, they cannot directly test for the presence of mediation effects, nor can they comprehensively assess the overall model-data fit in the way SEM does. In summary, although SCM and DAG hold significant value in the field of causal inference, given the theoretical framework and data conditions of this study, SEM was selected as the more scientifically sound and appropriate methodological choice due to its capacity for handling latent variables, controlling measurement error, and directly testing mediation pathways.

### 5.2 Model path testing

**Figure 3:**
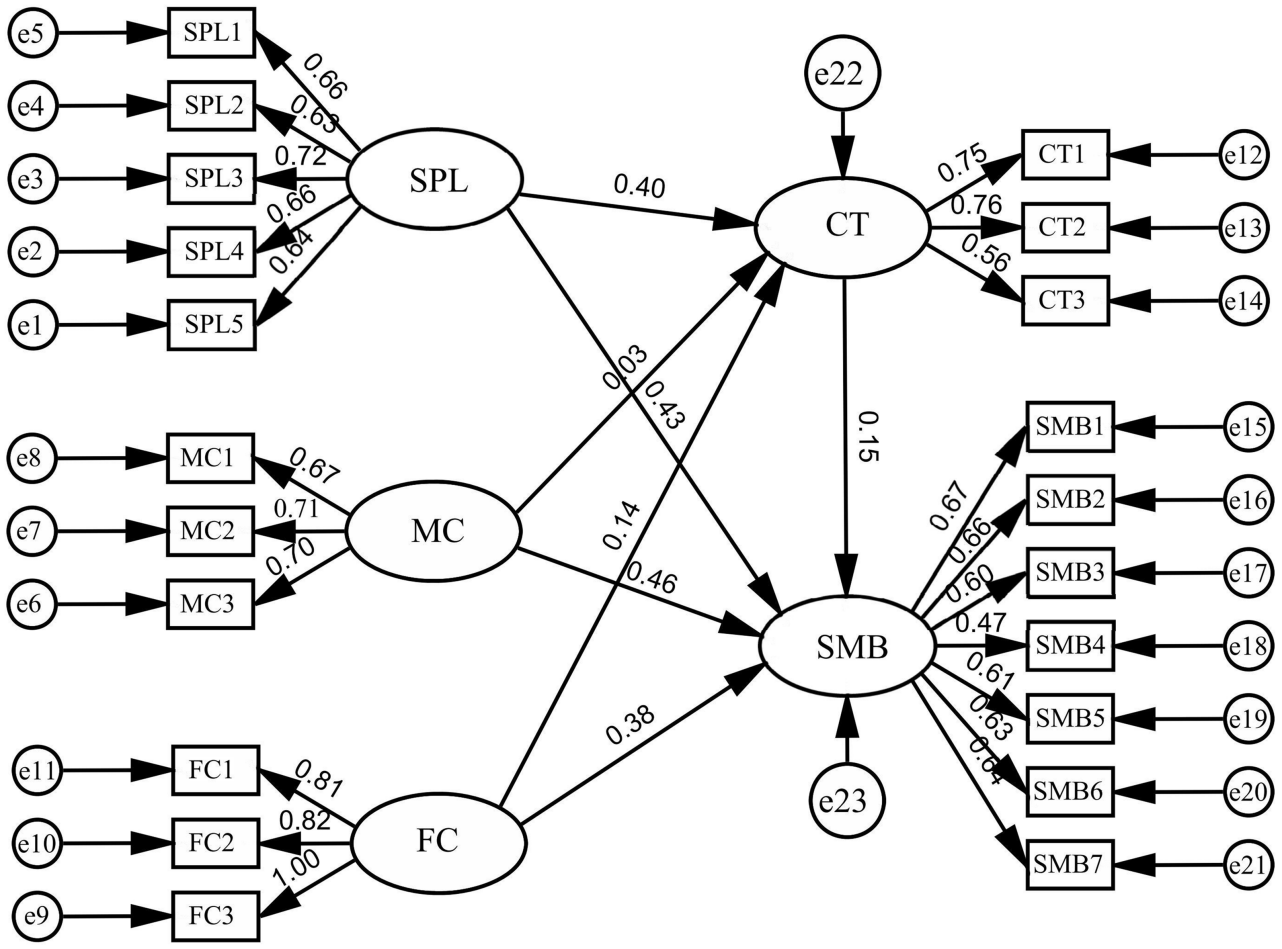
Model of factors influencing self-management behavior in older adult patients with chronic diseases.

The hypothesized model of factors influencing self-management behavior related to health science popularization short videos usage among older adult patients with chronic diseases is shown in Figure 3. The goodness-of-fit indices for the path analysis model are presented in Table 4. All indices fell within acceptable ranges, indicating a well-fitting model. The path analysis results are shown in Table 5. Self-perception level positively influenced content trustworthiness (*β* = 0.274, *p* < 0.001), supporting Hypothesis H1. A positive correlation was found between self-perception level and self-management behavior (*β* = 0.342, *p* < 0.001), supporting Hypothesis H2. Medical compliance positively influenced content trustworthiness (*β* = 0.219, *p* < 0.001), supporting Hypothesis H3. A positive correlation was found between medical compliance and self-management behavior (*β* = 0.284, *p* < 0.001), supporting Hypothesis H4. Family caregiving level positively influenced content trustworthiness (*β* = 0.251, *p* < 0.001), supporting Hypothesis H5. A positive correlation was found between family caregiving level and self-management behavior (*β* = 0.314, *p* < 0.001), supporting Hypothesis H6. Content trustworthiness positively influenced self-management behavior (*β* = 0.371, *p* < 0.001), supporting Hypothesis H7.

**Table 4:** Model fit results.

Indicator	CMIN/DF	RMSEA	CFI	IFI	RFI	TLI
Standard value	<3.0	<0.08	>0.90	>0.90	>0.90	>0.90
Actual value	2.873	0.056	0.939	0.939	0.902	0.914

**Table 5:** Results of path analysis.

Hypothetical	Pathway	Standardized path factorβ	*p*	Results
H1	SPL → CT	0.274	<0.001	Be tenable
H2	SPL → SMB	0.342	<0.001	Be tenable
H3	MC → CT	0.219	<0.001	Be tenable
H4	MC → SMB	0.284	<0.001	Be tenable
H5	FCL → CT	0.251	<0.001	Be tenable
H6	FCL → SMB	0.314	<0.001	Be tenable
H7	CT → SMB	0.371	<0.001	Be tenable

### 5.3 Mediation effect test

To examine whether content trustworthiness mediates the relationships between self-perception level, medical compliance, family caregiving level, and self-management behavior, the mediation model is shown on the right side of Figure 1. The indirect effect is denoted as ab and the direct effect as c’. Partial mediation is indicated when both ab and c’ are significant; complete mediation occurs when a*b is significant but c’ is not. This study employed the Bootstrap method for verification. Using AMOS 29.0, 2000 repeated samplings were performed. A significant mediation effect is confirmed if the bias-corrected nonparametric percentile 95% confidence interval does not include zero. The analysis results (Table 6) indicate that content trustworthiness fully mediates the relationship between self-perception level and self-management behavior, whereas it only partially mediates the relationships between medical compliance, family caregiving level, and self-management behavior. In summary, content trustworthiness serves as a mediating variable between the influencing factors and self-management behavior, thus supporting Hypothesis H8.

**Table 6:** Results of the mediation effect test.

Intermediary path	Effect type	Effect size	Bia-corrected 95% CI		Results
			Lower	Upper	
SPL → CT → SMB	Total effect	0.494	0.360	0.618	Full mediation
	Direct effect	0.182	−0.087	0.369	
	Indirect effect	0.062	0.025	0.111	
MC → CT → SMB	Total effect	0.462	0.340	0.563	Partial mediation
	Direct effect	0.457	0.332	0.557	
	Indirect effect	0.019	0.013	0.027	
FCL → CT → SMB	Total effect	0.405	0.299	0.495	Partial mediation
	Direct effect	0.383	0.282	0.476	
	Indirect effect	0.022	0.006	0.048	

## 6 Discussion and conclusion

### 6.1 Self-perception level, medical compliance, and family caregiving level jointly drive the self-management behavior of older adult patients with chronic diseases

The results indicate that the level of self-perception, medical compliance, and family caregiving are three significant factors influencing the self-management behavior of this population. Among these factors, self-perception level significantly promoted changes in self-management behavior. Path analysis revealed standardized path coefficients of 0.342 for self-perception, 0.284 for medical compliance, and 0.314 for family caregiving level (*p* < 0.001) on self-management behavior. This demonstrates that, within the context of health science popularization short videos, individuals’ disease perception, standardized engagement with internet-based healthcare, and support from family members serve as crucial intrinsic and extrinsic motivations for behavior change. These findings not only confirm the applicability of the Health Belief Model in a media-based management context for older adult patients with chronic diseases but also underscore the family’s role as a fundamental unit of social support that is pivotal in facilitating individual health behavior change (34, 35).

This phenomenon may stem from the synergistic interaction between individual health beliefs and new media exposure. Due to the protracted nature of their conditions and low self-efficacy, older patients often exhibit heightened selective attention to health information. Health science popularization short videos, leveraging their audiovisual and scenario-based communication strengths, translate professional medical knowledge into comprehensible visual content. By presenting clinical cases and demonstrating pathological processes, they effectively enhance older audiences’ perception of disease risks, thereby fostering a “mediatized risk perception. “This aligns closely with the “visual knowledge internalization” process observed by Bautista et al. in their study of diabetes health videos on YouTube, whereby audiovisual media bridge comprehension gaps caused by cognitive decline or low health literacy in older adults by concretizing complex pathologies (36). Older adult patients with chronic diseases with a higher level of self-perception can more proactively filter and trust short video content that matches their health needs, thereby facilitating a smoother translation of knowledge into self-management behavior (37). Medical compliance reflects patients’ trust in medical authority and behavioral inertia. This trust can be transferred to health science popularization short videos with professional endorsements, creating consistency from online viewing to offline action. The observed enhancement of medical compliance in this study underscores the potential of health short videos as a supplementary tool to traditional healthcare. This finding is consistent with research on Japanese hypertensive patients, which found that patients regularly following dance exercise videos on YouTube achieved significantly greater blood pressure reduction than a control group, demonstrating the efficacy of such videos over traditional exercise prescriptions and indirectly strengthening adherence to non-pharmacological advice like “regular exercise” (38). Family caregiving level provides not only emotional support but also, through “digital feedback” from younger generations, enhances older users’ media competency and confidence in content discernment. Behaviors such as children assisting with content filtering and elders proactively forwarding health videos reconstruct family health interaction rituals in digital space, strengthening intergenerational emotional bonds and a shared sense of health community. This collectively promotes the formation and maintenance of self-management behavior among older adult patients with chronic diseases at cognitive, behavioral, and emotional levels (39).

### 6.2 Content trustworthiness mediated the formation of health behaviors among older adult patients with chronic diseases

Bootstrap mediation tests confirmed the mediating role of content trustworthiness, a finding consistent with the work of Soroya et al. Content trustworthiness fully mediated the relationship between self-perception (*β* = 0.274) and self-management behavior, while it partially mediated the relationships of both medical compliance (*β* = 0.219) and family caregiving level (*β* = 0.251) with self-management behavior. Furthermore, content trustworthiness exerted a significant direct effect on self-management behavior (*β* = 0.371, *p* < 0.001). This indicates that trust in health science popularization short videos among older adult patients with chronic diseases serves not only as a psychological bridge for translating health communication effects but also as a key mechanism for converting individual cognition, medical intentions, and family support into practical health management. For the cognitive variable of self-perception, its influence on behavior change operates entirely through trust in media content. In contrast, medical compliance, as an established behavioral pattern, may be directly influenced by non-psychological factors such as the doctor-patient relationship, adherence to instructions, and long-term treatment habits, in addition to its indirect effect mediated by content trust (40). Similarly, while family caregiving level helps foster sustained trust in media information, it can also directly facilitate the implementation of self-management behavior through non-mediated pathways such as practical care, emotional encouragement, and behavioral supervision. Therefore, while communication strategies for health science popularization short videos should focus on building content trustworthiness through authoritative sources, scientific evidence, and rigorous narratives (41), the indispensable role of offline healthcare contexts and family support systems must also be acknowledged to develop more integrated health behavior interventions in the future. However, the flip side of this positive picture is the challenge of information quality in short videos. Particularly in the global context of rampant digital misinformation, if platform algorithms prioritize user engagement at the expense of tolerating exaggerated claims or unverified pseudoscience, they risk severely eroding the fragile trust between platforms and users, undermining users’ daily health decisions. Consequently, governmental policy-making and corporate platform governance must strive to foster an information ecosystem characterized by “algorithmic benevolence.” Measures may include prioritizing content from creators verified by authoritative health institutions, implementing tiered labeling for health videos, and enhancing rumor-refuting mechanisms tailored for older users, thereby curbing the spread of false content at its source and elevating the credibility of the information environment.

### 6.3 Disparities in geographic location (urban vs. rural) and media exposure reflect differences in digital health accessibility

The survey results indicate that the self-management behavior of rural residents was significantly lower than that of urban residents (*β* = −0.715, *p* < 0.05). This finding, consistent with Liu et al. (42), demonstrates lower digital participation and self-management behavior among older adult patients with chronic diseases in rural China. In contrast, users who frequently watch and actively engage with content from official institutions or medical self-media platforms show more positive behavioral changes. This urban–rural disparity likely stems from inequalities in healthcare resource availability, health information service coverage, digital access infrastructure, and educational attainment. Older adult patients with chronic diseases in rural areas often face multiple challenges, including insufficient primary healthcare services, difficulties using smart devices, and a lack of digital feedback (43). These constraints can lead to cognitive confusion, information overload, or avoidance during content processing. Therefore, when advancing digital public health communication, it is crucial to provide technical support and prioritize digital inclusion for rural and older populations with chronic diseases. Digital media platforms should also proactively integrate elder-friendly principles and age-appropriate design into their products to prevent the widening of the “health gap” (44). Furthermore, older adults in rural areas rely more heavily on interpersonal communication as a primary information channel. The emotional support and immediate interaction from family and neighbors often influence them more profoundly than purely scientific persuasion (45). Thus, relying solely on independent viewing of short videos is insufficient for significantly improving their health management; it requires external support through digital feedback and family interaction. For instance, family caregivers and healthcare providers can actively assist older patients in filtering and interpreting high-quality short video content while also integrating the knowledge gained online into the patient’s disease care and medical instruction communication. This collaborative approach can promote the effective translation of health information and advance the goal of social health equity.

Analysis of mean values indicates that older adult patients with chronic diseases who watch short videos more frequently and for longer durations demonstrate significantly more pronounced self-management behavior (*p* < 0.001). High-frequency and prolonged exposure to health science popularization short videos helps enhance patients’ familiarity with disease knowledge, reduces cognitive load, and assists them in developing a systematic understanding of health issues through diverse content and social interaction. However, factors such as platform culture, content creator profiles, and user media literacy contribute to a partial communication “gap” between users and healthcare professionals. Consequently, specific diagnostic inquiries and practical needs may not be adequately integrated into the content creation process (46). Driven by algorithmic recommendation mechanisms, sustained use of health videos will enable the platform to gradually optimize and push content that matches an individual’s health needs, creating an increasingly personalized and accurate information environment tailored to individual health needs. More importantly, participatory behaviors such as commenting and sharing transform older adults from passive information recipients into active participants within health communication networks. This social interaction and emotional support strengthen their confidence and motivation for behavior change (47). However, this advantage is accompanied by limitations common to global short-video platforms. Similar to the risks of information oversimplification and lack of evidence often observed in “health challenge” trends on TikTok, this study finds that for older users, algorithm-driven “information cocoons” can reinforce their existing cognitive biases. Prolonged immersion in homogeneous information streams may not only fail to enhance critical health literacy but could also reduce their motivation to proactively seek diverse and authoritative information sources. This suggests that merely increasing media exposure frequency is insufficient for fostering informed health citizens if platforms lack effective content quality regulation and value-guided algorithms. Instead, it may exacerbate cognitive biases and create new “knowledge gaps” among older patients. Therefore, it is crucial to strengthen the governance of content scientific rigor and enhance algorithmic transparency within platform operations.

Furthermore, no significant difference in self-management behavior was observed based on gender among older adult patients with chronic diseases. Similarly, education level and income did not exert a direct impact on content trustworthiness. This indicates that the formation of trust in media relies more on individual cognitive and affective factors than on educational attainment or economic status (11). In health communication, the perceived credibility of the content, emotional resonance, and social identity are more potent drivers of trust among older users than traditional socio-economic factors. Consequently, future health communication strategies should focus more on enhancing content persuasiveness, securing endorsements from authoritative institutions, and leveraging interpersonal networks for diffusion, rather than targeting only highly educated or affluent demographic groups.

## 7 Theoretical and practical implications

Theoretically, this study enriches the application scenarios of the Health Belief Model and extends the explanatory boundaries of health behavior and social support theories within the context of digital media. It constructs and verifies a core pathway of “media usage → content trust → behavior change,” revealing the moderating role of family caregiving level and digital feedback in trust formation. This provides interdisciplinary theoretical support for health communication research targeting older adults. On a practical level, previous health science popularization efforts have often prioritized information accuracy and accessibility, while overlooking the emotional needs of the older adult population and urban–rural resource disparities. This study offers content design strategies that balance authoritative information with emotional resonance for health science popularization short videos. It emphasizes the buffering role of intergenerational digital feedback in compensating for insufficient grassroots community services. The findings also provide a significant reference for developing future health interventions for older adult patients with chronic diseases and for promoting public health communication and digital inclusion in an aging society.

## 8 Conclusion

The empirical results of this study demonstrate that the use of health science popularization short videos exerts a significant positive influence on the self-management behavior of older adult patients with chronic diseases in China. Self-perception level, medical compliance, and family caregiving level were identified as key factors influencing the development of self-management behavior through social media use among this population. Content trustworthiness plays a mediating role between the use of health science popularization short videos and self-management behavior. The formation of this media trust was found to be independent of the users’ educational attainment or economic status. These findings underscore the importance of several factors for achieving better health outcomes: enhancing the health literacy of older patients, improving the information quality of health science popularization short videos, bridging the urban–rural digital divide, and fostering digital inclusion, intergenerational support, and emotional care.

## 9 Limitations of the study

This study has several limitations. First, despite employing multi-stage stratified sampling, the proportion of rural respondents remained relatively low. Future research should expand the sample size to enhance the representation of rural, advanced-age, and low socioeconomic status populations, thereby providing a more robust basis for policy formulation. Second, factors influencing self-management behavior are diverse and complex. While this study focused on five core variables, it did not incorporate other potential factors such as platform algorithms, the information cocoon effect, or baseline health literacy. The comprehensiveness of the variable framework could be improved. Third, as a cross-sectional study, it can reveal correlations and mediating mechanisms among variables but cannot capture the long-term effects of health science popularization short videos use or the developmental trajectory of patients’ self-management behavior. Fourth, the reliance on self-reported data collected via electronic questionnaires means that memory bias and reporting inaccuracies cannot be entirely ruled out. Future studies could incorporate objective behavioral indicators for multi-source data triangulation. In conclusion, subsequent research should build upon an expanded sample size to investigate the unexamined potential factors, employ longitudinal designs, and collect multi-source data to more precisely assess the long-term impact of health science popularization short videos on the self-management behavior of older adult patients with chronic diseases. Despite these limitations, the findings contribute positively to current efforts aimed at fostering sustainable healthy lifestyles through digital health knowledge, refining smart aging policy systems, and constructing an age-friendly health communication ecosystem.

## Data Availability

The original contributions presented in the study are included in the article/Supplementary material, further inquiries can be directed to the corresponding author/s.

## Footnotes

1
https://www.credamo.com/
