# Supplementary material for: Research on the influencing factors of using health science popularization short videos on the self-management behavior of older adult patients with chronic diseases
Source: Front Public Health. 2026 Jan 21;14:1720548. doi: 10.3389/fpubh.2026.1720548 (PMC12871061; doi:10.3389/fpubh.2026.1720548)
Supplement: Supplementary file 1 [file Data_Sheet_1.docx]

**Supplementary Material: Questionnaire**

Dear Participant,

Greetings. I am a graduate student at Qingdao University of Technology, currently conducting academic research on the impact of health science short video usage on the self-management behavior of older adult patients with chronic diseases. This questionnaire aims to understand your experiences and perceptions regarding digital media use. It will take approximately 5 to 8 minutes to complete. Participation is entirely voluntary. You have the right to withdraw at any time without any adverse consequences. This survey is anonymous. All your responses will be strictly confidential and used solely for aggregate statistical analysis. No personally identifiable information will appear in any reports. All electronic data will be stored in encrypted files. Please feel assured in providing your responses. Should you have any questions regarding this study, please do not hesitate to contact me via email at 1143692974@qq.com.

Note: "Health science short videos" refer to short video clips, typically lasting from seconds to tens of seconds, primarily designed to disseminate health knowledge and serve a public science education purpose. "Chronic diseases" refer to conditions characterized by long duration, slow progression, and difficulty in cure. Common types include, but are not limited to, cardiovascular diseases (e.g., hypertension, coronary heart disease), diabetes, chronic respiratory diseases, and cancers, encompassing one or multiple such conditions.

If you agree with the above information and voluntarily consent to participate in this study, please check the box below and proceed to the questions.

□I have read and understood the above information, and I voluntarily agree to participate in this study.

**Section I: Demographic Information**

1. What is your gender?

A. Male

B. Female

2. What is your age?

A. 60-64

B. 65-69

C. 70-74

D. 75-79

E. ≥80

3. What is your type of residence?

A. Rural

B. Urban

4. What is your highest level of education?

A. Primary school or below

B. Junior high school

C. Senior high school

D. Junior college

E. Bachelor's degree or above

5. What is your average personal monthly income?

A. No fixed income

B. <1000

C. 1000–3000

D. 3001–5000

E. ≥5000

**Section II: Social Media Usage**

6. How frequently do you typically watch health science short videos?

A. <1/month

B. 1-3/month

C. 1-2/week

D. 3-6/week

E. ≥1/day

7. On average, how much time do you spend daily watching health science short videos?

A. <0.5h

B. 0.5–1h

C. 1–2h

D. ≥2h

8. On which platforms do you typically watch health science short videos? (Select all that apply)

A. Public social media (e.g., WeChat Channels, Weibo, TikTok, Kuaishou)

B. Official social media (e.g., People's Daily, CCTV News, local healthcare institution apps)

C. Content aggregation platforms (e.g., Toutiao, Baidu Browser)

D. Medical and health self-media (e.g., Dingxiang Doctor, Haodf)

9. What types of health information do you typically focus on when watching these videos? (Select all that apply)

A. Exercise, healthy diet, and lifestyle habits

B. Mental health and emotional management

C. Disease symptoms, diagnosis, and treatment

D. Others' illness experiences and solutions

E. Drug information

F. Medical insurance and health policies

G. Doctor or healthcare institution information

H. Other (Please specify:______)

10. Which of the following chronic conditions do you have? (Select all that apply)

A. Hypertension, Hyperlipidemia

B. Coronary heart disease, Chronic heart failure, Heart disease

C. Diabetes, Hyperuricemia

D. Emphysema, Asthma, Bronchitis

E. Fatty liver, Cirrhosis

F. Arthritis, Rheumatoid disease, Lumbar disc herniation, Cervical spondylosis

G. Chronic gastritis, Chronic enteritis, Peptic ulcer

H. Other (Please specify:______)

11. To what extent do you believe watching health science short videos has impacted the management of your chronic disease(s)?

A. Very helpful, with a noticeable effect

B. Somewhat helpful, but the effect is limited

C. Not very helpful, I primarily manage my condition through other means

D. Not helpful at all

**Section III: Self-Management Behavior Scale**

Please indicate your level of agreement with each of the following statements based on your personal experience by selecting the corresponding number.

1 = Strongly disagree; 2 = Disagree; 3 = Neutral; 4 = Agree; 5 = Strongly agree

| Serial number | Question | Option | | | | |
| --- | --- | --- | --- | --- | --- | --- |
|  |  | Strongly disagree | Disagree | Neutral | Agree | Strongly agree |
| 13 | After watching health science popularization short videos, I perceive myself as susceptible to complications or other health issues related to chronic diseases. | 1 | 2 | 3 | 4 | 5 |
| 14 | After watching health science popularization short videos, I recognize that my usual unhealthy lifestyle habits pose a threat to my health. | 1 | 2 | 3 | 4 | 5 |
| 15 | Through watching health science popularization short videos, I have gained better understanding of chronic diseases and intend to pay more attention to my health issues in the future. | 1 | 2 | 3 | 4 | 5 |
| 16 | I find that watching health science popularization short videos helps alleviate my disease-related tension and anxiety. | 1 | 2 | 3 | 4 | 5 |
| 17 | I consider the content in health science popularization short videos to be clearly presented and have not encountered information that is difficult to comprehend. | 1 | 2 | 3 | 4 | 5 |
| 18 | After watching health science popularization short videos, I proactively seek medical help from doctors. | 1 | 2 | 3 | 4 | 5 |
| 19 | After watching health science popularization short videos, I actively consult about my condition by leaving comments in online sections or sending private messages to doctors. | 1 | 2 | 3 | 4 | 5 |
| 20 | With reminders and supervision from family or friends, I follow the recommendations in videos for self-monitoring, regular check-ups, and cooperation with healthcare providers. | 1 | 2 | 3 | 4 | 5 |
| 21 | My children/grandchildren regularly initiate sharing health science popularization short videos with me. | 1 | 2 | 3 | 4 | 5 |
| 22 | My children/grandchildren show concern about my condition and guide me in using smartphones to watch health science popularization short videos. | 1 | 2 | 3 | 4 | 5 |
| 23 | My children/grandchildren also remind me to be cautious about potential misinformation in health science popularization short videos. | 1 | 2 | 3 | 4 | 5 |
| 24 | I am concerned that inaccurate information in health science popularization short videos from non-official media may affect my disease treatment. | 1 | 2 | 3 | 4 | 5 |
| 25 | I am skeptical about the content of health science popularization short videos released by non-official media. | 1 | 2 | 3 | 4 | 5 |
| 26 | I consider health science popularization short video content recommended or published by authoritative channels to be reliable. | 1 | 2 | 3 | 4 | 5 |
| 27 | After watching health science popularization short videos, I actively search for additional online information about chronic disease treatment. | 1 | 2 | 3 | 4 | 5 |
| 28 | I share the health knowledge gained from popularization short videos with family or friends. | 1 | 2 | 3 | 4 | 5 |
| 29 | After watching health science popularization short videos, I have gradually adjusted my daily routine, dietary habits, medication adherence, and increased physical exercise. | 1 | 2 | 3 | 4 | 5 |
| 30 | I proactively verify health science short video content about which I am skeptical and make rational healthcare decisions based on my specific condition. | 1 | 2 | 3 | 4 | 5 |
| 31 | After watching health science popularization short videos, I have significantly increased the frequency and duration of my daily exercise. | 1 | 2 | 3 | 4 | 5 |
| 32 | After watching health science popularization short videos, I feel more confident in managing my chronic condition. | 1 | 2 | 3 | 4 | 5 |
| 33 | I am willing to continue watching similar health science videos to support my health management. | 1 | 2 | 3 | 4 | 5 |

We sincerely thank you for your valuable time in completing this survey. We wish you continued success in your health management practices and ongoing health and well-being.
